# The splenial angle: a novel radiological index for idiopathic normal pressure hydrocephalus

**DOI:** 10.1007/s00330-021-07871-4

**Published:** 2021-05-15

**Authors:** Ling Ling Chan, Robert Chen, Huihua Li, Amanda J. Y. Lee, Wei Ying Go, Weiling Lee, Christine Lock, Sumeet Kumar, Adeline S. L. Ng, Nagaendran Kandiah, Louis C. S. Tan, Eng King Tan, Nicole C. H. Keong

**Affiliations:** 1 Diagnostic Radiology, Singapore General Hospital, Outram Road, Singapore, 169608 Singapore; 2 Duke-NUS Medical School, Singapore, Singapore; 3 Health Services Research Unit, Singapore General Hospital, Singapore, Singapore; 4 Neurosurgery, National Neuroscience Institute, Singapore, Singapore; 5 Neuroradiology, National Neuroscience Institute, Singapore, Singapore; 6 Neurology, National Neuroscience Institute, Singapore, Singapore

**Keywords:** Hydrocephalus, normal pressure, Neurodegenerative diseases, Magnetic resonance imaging, Diagnosis, differential, ROC curve

## Abstract

**Objectives:**

To evaluate the utility of the splenial angle (SA), an axial angular index of lateral ventriculomegaly measured on diffusion tensor MRI color fractional anisotropy maps, in differentiating NPH from Alzheimer’s disease (AD), Parkinson’s disease (PD), and healthy controls (HC), and post-shunt changes in NPH, compared to Evans’ index and callosal angle.

**Methods:**

Evans’ index, callosal angle, and SA were measured on brain MRI of 76 subjects comprising equal numbers of age- and sex-matched subjects from each cohort of NPH, AD, PD, and HC by two raters. Receiver operating characteristics (ROC) and multivariable analysis were used to assess the screening performance of each measure in differentiating and predicting NPH from non-NPH groups respectively. Temporal changes in the measures on 1-year follow-up MRI in 11 NPH patients (with or without ventriculoperitoneal shunting) were also assessed.

**Results:**

Inter-rater and intra-rater reliability were excellent for all measurements (intraclass correlation coefficients > 0.9). Pairwise comparison showed that SA was statistically different between NPH and AD/PD/HC subjects (*p* < 0.0001). SA performed the best in predicting NPH, with an area under the ROC curve of > 0.98, and was the only measure left in the final model of the multivariable analysis. Significant (*p < *0.01) change in SA was seen at follow-up MRI of NPH patients who were shunted compared to those who were not.

**Conclusions:**

The SA is readily measured on axial DTI color FA maps compared to the callosal angle and shows superior performance differentiating NPH from neurodegenerative disorders and sensitivity to ventricular changes in NPH after surgical intervention.

**Key Points:**

*• The splenial angle is a novel simple angular radiological index proposed for idiopathic normal pressure hydrocephalus, measured in the ubiquitous axial plane on DTI color fractional anisotropy maps.*

*• The splenial angle quantitates the compression and stretching of the posterior callosal commissural fibers alongside the distended lateral ventricles in idiopathic normal pressure hydrocephalus (NPH) using tools readily accessible in clinical practice and shows excellent test-retest reliability.*

*• Splenial angle outperforms Evans’ index and callosal angle in predicting NPH from healthy, Parkinson’s disease, and Alzheimer’s disease subjects on ROC analysis with an area under the curve of > 0.98 and is sensitive to morphological ventricular changes in NPH patients after ventricular shunting.*

**Supplementary Information:**

The online version contains supplementary material available at 10.1007/s00330-021-07871-4.

## Introduction

Idiopathic normal pressure hydrocephalus (NPH) is characterized by the classic triad of gait apraxia, dementia, and urinary incontinence but confounding factors commonly exist [1, 2]. Neuroimaging provides supplementary information to the comprehensive clinical assessment of NPH [3–5]. Ventriculomegaly in the absence of obstructive hydrocephalus or raised intracranial pressure is typical. Specifically, disproportionately enlarged subarachnoid space hydrocephalus, characterized by ventriculomegaly with sulcal crowding near the vertex and enlargement of the Sylvian fissures, is well described [4]. Evans’ index (EI), a quantitative radiological index included in the international and Japanese guidelines for the diagnosis and management of NPH [3–5], is a well-accepted gross measure of ventriculomegaly. However, it is non-specific, performs poorly at distinguishing between differing causes of ventriculomegaly, and is an unreliable predictor for shunt responsiveness [6].

The callosal angle (CA) is more specific and useful in discriminating NPH from other neurodegenerative disorders with overlapping clinico-radiological presentations of ventriculomegaly from brain atrophy, cognitive impairment, and/or Parkinsonism gait disorder [4, 5]. A narrow CA in NPH is believed to result from mechanical stretching of the corpus callosum by enlarged lateral ventricles and compression by enlarged Sylvian fissures. A small CA is also associated with better shunt responses [4, 7, 8]. Yet, given its advantages, the CA measurement is limited by its notable variability in practice [7, 9].

**Fig. 1:**
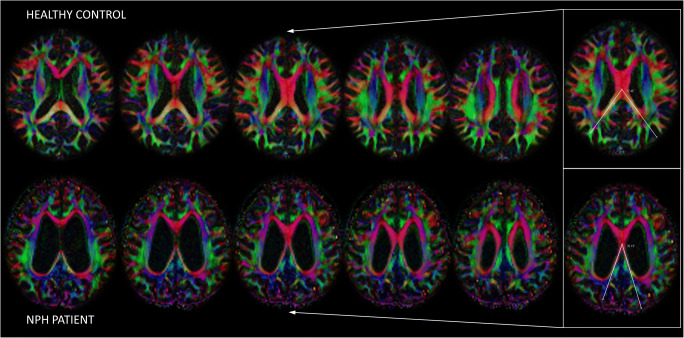
Consecutive axial diffusion tensor imaging (DTI) fractional anisotropy (FA) maps in a healthy control subject and a patient with idiopathic normal pressure hydrocephalus (NPH) demonstrating gross deformation of the red-encoded callosal commissural fibers, and depiction of the splenial angle (SA) as a simple angular index (inset) of the compression and stretching of the posterior callosal commissural fibers alongside the posterior-medial walls of the lateral ventricles. The SA is placed over the limbs of the forceps major and pivoted over the midline on the first axial slice containing the complete body of the corpus callosum. Note the severely narrowed SA in the NPH patient arising from the gross ventricular distension compared to that in the HC

Diffusion tensor imaging (DTI) fractional anisotropy (FA) maps are increasingly incorporated into clinical practice [10, 11] due to their rapid acquisition and availability on commercial DTI software packages. The unique anatomical information afforded by its color encoding for directional molecular water diffusion allows for consistent identification of major fiber tracts. In NPH, gross deformation of the red-encoded callosal commissural fibers is well depicted on color FA maps (Fig. 1). We propose the splenial angle (SA) as a novel alternative axial angular index of lateral ventricular dilatation for the clinical workup of NPH. We hypothesize that the SA could quantitate lateral ventricular distension in NPH as the CA does. We evaluated its (i) values, reproducibility, and ease of training; (ii) temporal changes in NPH patients after shunt surgery; and (iii) performance in differentiating NPH from Alzheimer’s disease (AD), Parkinson’s disease (PD), and healthy control (HC), compared to EI and CA.

## Materials and methods

Approval from the institutional ethics committee for each NPH, AD, and PD study was obtained. All participants gave informed consent.

### Clinical

Twenty patients were prospectively recruited from the NPH program at a tertiary referral center. All patients were diagnosed based on key clinical and neuroimaging features according to established criteria in published international NPH guidelines [3, 12], including supplementary neuropsychological testing and confirmation with cerebrospinal fluid (CSF) drainage using an in-house protocol adapted from the guidelines [3, 4, 13]. Patients deemed unlikely to have NPH or fit the diagnostic criteria for AD, PD, and/or had response to levodopa were excluded. All NPH patients demonstrated communicating hydrocephalus with ventriculomegaly (EI ≥ 0.30 or a bicaudate index ≥ 0.25) and included patients who met the guidelines’ classification for probable NPH (had ≥ two out of three features of the NPH triad) and possible NPH (had (a) incontinence and/or cognitive impairment in the absence of an observable gait/balance disturbance, or (b) gait disturbance/dementia alone) categories. Responsiveness to CSF drainage was assessed using a 10-m walking test, Tinetti gait and balance examinations, and a mini-mental state examination. Patients deemed to be responders were offered surgical intervention in the form of ventriculoperitoneal shunting. One patient who showed a large suprasellar cisternal cyst in addition to typical NPH features on brain MRI was excluded from this retrospective imaging study. Nineteen NPH patients were included in the final analysis. Longitudinal changes to the MRI measurements were also recorded on follow-up brain MRI scans available at 1 year.

Anonymized brain MRI of nineteen subjects each from the AD, PD, and HC databases age- and sex-matched to the nineteen NPH patients were sought for inclusion in this imaging analysis. The AD and PD patients were diagnosed based on established clinical criteria and recruited from the memory and movement disorders clinics respectively of a tertiary referral center [14–16]. AD was diagnosed using the revised National Institute of Neurological and Communicative Disorders and Stroke-Alzheimer’s Disease and Related Disorders Association criteria [17] with exclusion criteria comprising clinical evidence of stroke, Parkinsonism, NPH, serious systemic diseases, and psychiatric disorders. PD diagnosis was as defined by the Advisory Council of the United States National Institute of Neurological Disorders and Stroke [18], with the following exclusion criteria: cognitive and/or musculoskeletal dysfunction, features suggestive of stroke, NPH, Parkinson-plus syndromes, and secondary parkinsonism. The age- and sex-matched controls were healthy without any known neurodegenerative diseases.

### Brain MRI

For the NPH cohort, brain MRI was performed on a 3-T scanner including the following sequences: (A) fluid-attenuated inversion-recovery (FLAIR) (voxel size 0.8 × 0.8 × 4 mm^3^; repetition time ms/echo time ms, 7700/134; plane, axial), (B) three-dimensional (3D) T1-weighted high-resolution magnetization-prepared rapid gradient-echo (MPRAGE) (0.9 × 0.9 × 0.9 mm^3^; 1900/2.48; coronal) and (C) DTI (1.7 × 1.7 × 2 mm^3^; 10,025/97; *b* values 1000 and 0 s/mm^2^, 20 directions; acquisition time, 7:43 min; axial). All axial acquisitions were parallel to the central inter-commissural line through the anterior and posterior commissures (AC-PC) identified by the MRI technologists on sagittal scout images as per institutional clinical routine for more than two decades of our MRI service. NPH patients with follow-up brain MRI at 1-year from baseline scan were performed using an identical protocol on the same 3-T scanner.

Brain MRI of the AD, PD, and HC subjects were all acquired on 3-T scanners using similar MR protocols with the following parameters: (A) FLAIR (0.9-1.0 × 0.9-1.0 × 1-4 mm^3^; 5000-7150/74-387; axial), (B) 3D MPRAGE (0.9-1.0 × 0.9-1.0 × 0.9-1.0 mm^3^; 1900-2300/2.28-3; sagittal), and (C) DTI (1.8-2.3 × 1.8-2.3 × 2-2.5 mm^3^; 5600-10,118/54-102; *b* values 800-1000 and 0 s/mm^2^, 30-61 directions; 9:32-11:09 min; axial). All axial acquisitions were parallel to the AC-PC line as above.

### Image analysis

**Fig. 2:**
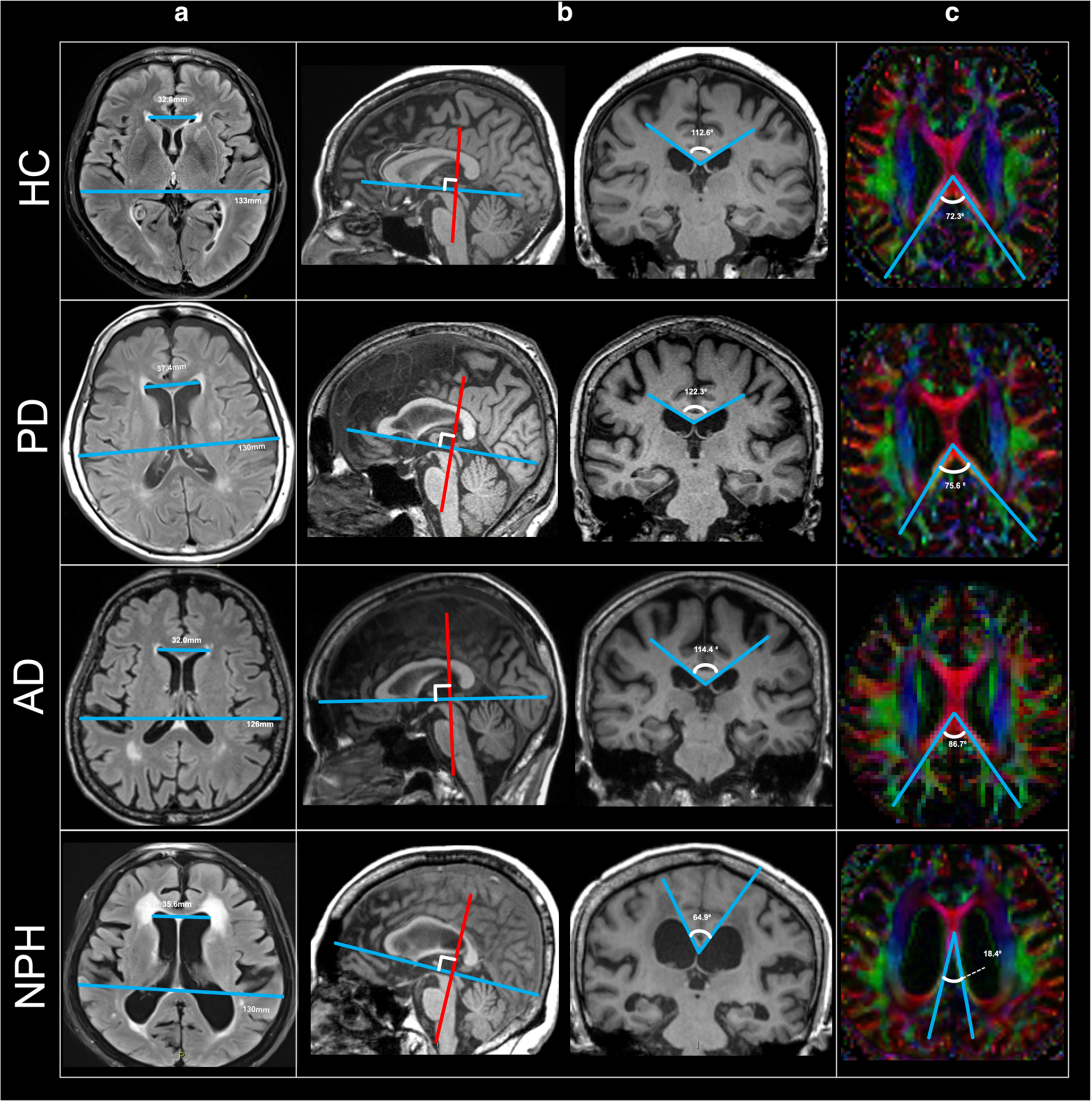
Evans’ index (EI), callosal angle (CA), and splenial angle (SA)measured on brain MRI in healthy controls (HC), Parkinson’s disease (PD), Alzheimer’s disease (AD), and normal pressure hydrocephalus (NPH) patients. **a** Axial FLAIR images demonstrating EI as a ratio of the maximal width of the frontal horns of the lateral ventricles (short blue line) against the maximal internal diameter of the cranium (long blue line) on the same section. **b** Left sagittal images depicting the coronal plane (red line) orthogonal to the anterior-posterior commissural (AC-PC) plane (blue line), and right coronal images demonstrating the CA subtended against the roof of the lateral ventricles. **c** Axial diffusion tensor imaging (DTI) color FA images containing the complete red-encoded callosal body depicting the SA subtended over the limbs of the forceps major and pivoted over the midline. Note the severely narrowed SA in an NPH patient arising from deformation of the red-encoded posterior callosal commissural fibers secondary to gross ventricular distension

Image analysis was performed on the brain MRIs of 76 subjects comprising 19 NPH patients, and equal numbers each of AD, PD, and HC subjects. EI, CA, and SA measurements (Fig. 2) were made by 2 blinded independent raters: a neuroradiologist with more than 20 years of experience and a radiology research assistant. The EI [3–5] (Fig. 2a) was measured as the maximal width of the frontal horns of the lateral ventricles to the maximal internal diameter of the cranium on the same axial FLAIR image. The CA [7, 8] (Fig. 2b) was measured as the angle subtended by the roof of both lateral ventricles on a coronal MPRAGE image through the PC. Care was undertaken, during multiplanar reformatting on the 3D high-resolution MPRAGE series, to ensure that the coronal image upon which the CA was measured was truly orthogonal to the AC-PC plane as determined on multiplanar views.

Using the DTI color FA maps, the SA was measured on the first axial slice containing the complete body of the corpus callosum encoded in red, when scrolling in a caudocranial direction (Figs. 1 and 2c). The SA is an angular quantitative measure of the compression and stretching of the red-encoded posterior callosal commissural fibers alongside the posterior-medial walls of the lateral ventricles. The angle is pivoted over the midline with its arms aligned along the center of limbs of the forceps major. The forceps major is more severely compressed by the ventricular distension than the forceps minor in NPH patients (Fig. 1), and as a result, the angular pivot of the SA thus measured lies farther anteriorly in NPH patients than AD, PD, and HC subjects (Fig. 2c). In subjects where the thicker callosal body is completely imaged on 2 slices, the caudal image is used for the SA measurement.

### Statistical analysis

Data analysis was performed using R 3.6.2 (https://www.r-project.org). Intra-rater and inter-rater reliability were assessed by intraclass correlation coefficients (ICC) (single measurement, absolute agreement, 2-way mixed-effects model) for all measurements. The averaged measurements from the two raters were used for each group comparison (since the ICC indicated excellent inter- and intra-rater reliability). Fisher’s exact test for categorical variables and ANOVA test for continuous variables were used to compare the demographic data and MRI brain measurements. In view of the multiple comparisons made, pairwise comparison of EI, CA, and SA adjusted by the Benjamini and Hochberg method was carried out to compare differences in these measurements between HC, PD, AD, and NPH groups by controlling the false discovery rate, the latter being the expected proportion of false discoveries amongst the rejected null hypotheses. Receiver operating characteristics (ROC) analysis was carried out to evaluate the sensitivity and specificity of the three indices as screening diagnostic tools for NPH from each non-NPH group individually. The area under the curve (AUC) highlighted the discriminative property of each index. A stepwise multivariable logistical regression analysis was performed to predict NPH from non-NPH groups with brain MRI measures as independent factors. Student’s *t* test was carried out to compare the changes from baseline in EI, CA, and SA between NPH patients who underwent ventriculoperitoneal shunting and those who did not. All statistical tests were evaluated as two-sided tests and statistical significance was defined at *p* < 0.05.

## Results

The demographics of the 76 participants in the four groups and the mean and standard deviation values of their brain MRI measurements are shown in Table 1, with boxplots of the group measurements graphically depicted in Fig. 4. The cohorts were well matched for age and sex. While the NPH patients met the EI criterion of ≥ 0.3, the HC group had EI well within the normal cutoff of < 0.3, and the EI of the PD and AD patients straddled across 0.3. Similar to the CA, the SA range was narrower (mostly < 35°) and distinct from the non-NPH groups (mostly > 45°) in general (see Supplementary Materials, Table 1 EI/CA/SA ranges and CA-SA scatterplot figure).

**Table 1:** Demographics of case-control study subjects and mean quantitative brain MRI measures

Characteristic	HC	PD	AD	NPH	*p* value
No. of patients	19	19	19	19	
Mean age (years ± SD)	72.3 ± 5.38	73.6 ± 5.74	73.8 ± 5.60	73.7 ± 6.36	0.825
No. of men	11	11	11	11	1.000
No. of women	8	8	8	8	
Mean MRI measure + SD					
EI	0.25 ± 0.02	0.29 ± 0.05	0.3 ± 0.06	0.38 ± 0.08	**< 0.0001**
CA (°)	112 ± 8.05	105 ± 20.0	104 ± 20.3	58.3 ± 15.6	**< 0.0001**
SA (°)	75.7 ± 13.1	67.0 ± 18.8	70.5 ± 11.9	25 ± 9.74	**< 0.0001**

*Note: HC* healthy controls, *PD* Parkinson’s disease, *AD* Alzheimer’s disease, *NPH* idiopathic normal pressure hydrocephalus, *EI* Evans’ index, *CA* callosal angle, *SA* splenial angle

The ANOVA test was used for comparison of the MRI measurements between groups, with statistical significance defined at *p* < 0.05 (marked in bold)

The inter-rater reliability was excellent for all measurements and detailed for each measurement and group in Supplementary Table 2. The ICCs and 95% confidence intervals for EI were 0.97 (0.93, 0.99), CA 0.99 (0.99, 1.00), and SA 0.99 (0.98, 0.99). Intra-rater reliability was also excellent with ICCs > 0.90 for EI, CA, and SA measurements repeated for a subset of 38 subjects by the research assistant after a time-lapse of 1 month.

**Fig. 3:**
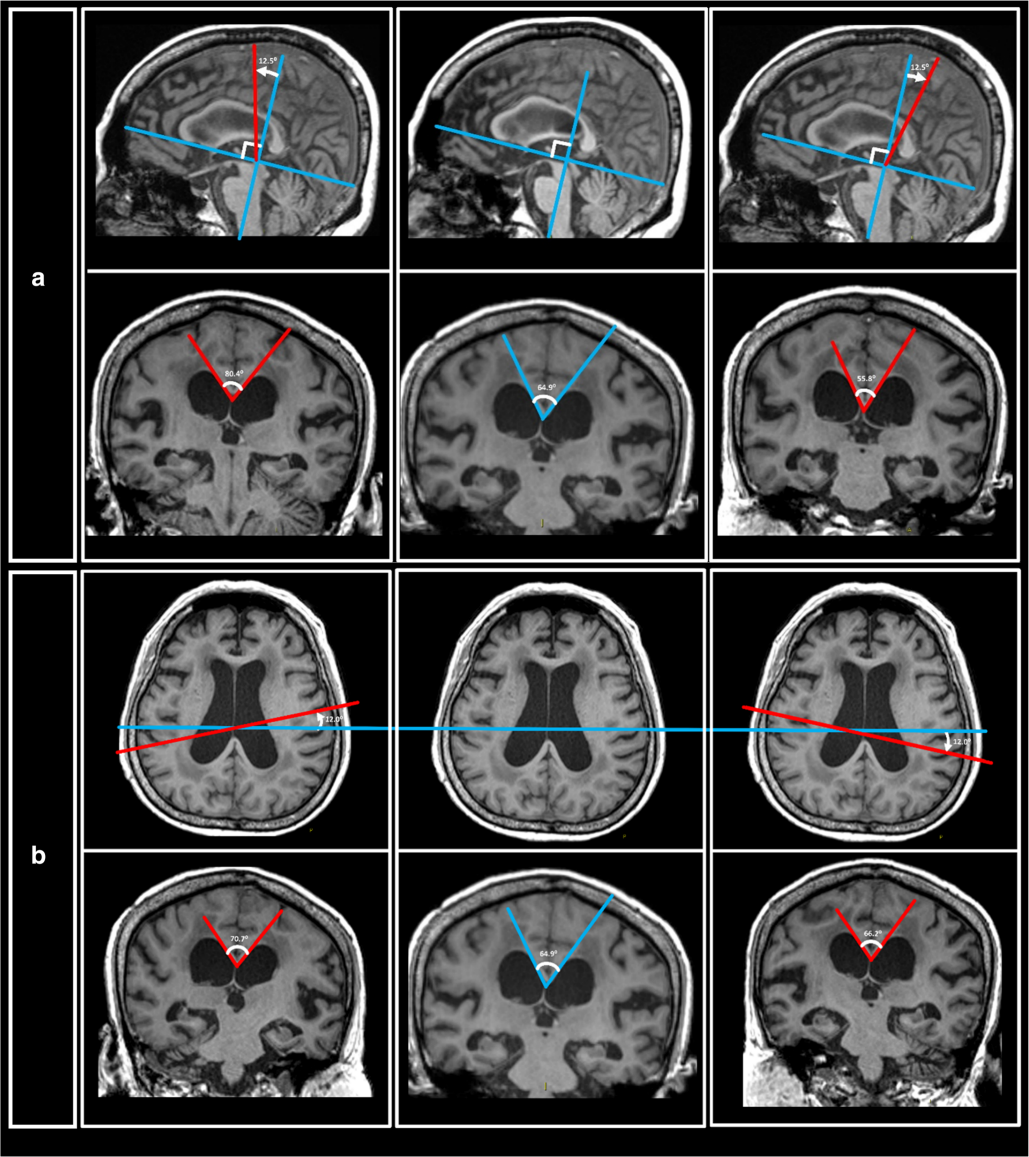
Potential confounders to accurate, reproducible callosal angle (CA) measurements. Multiple pitfalls exist to positioning a coronal plane truly orthogonal to the anterior commissure-posterior commissural (AC-PC) plane for CA measurements. Greater variability in the CA measurement occurs with **a** erroneous acute/obtuse angulation of the coronal plane (CA ranged from 55.8° (right) to 80.4° (left) compared to 64.9° (middle) on the “true” coronal plane) than with **b** lateral right-left malrotations (CA was inconsistently variable: 66.2° (right) to 70.7° (left) compared to 64.9° (middle) on the “true” coronal plane), compared to the ease of SA measurement on a ubiquitous axial plane of acquisition (without need for plane reformation). These potential pitfalls make CA measures clinically less reproducible, limiting its ease of utility as a reliable imaging biomarker

Both the EI and SA measurements were easy to train in. The research assistant readily acquired proficiency in effecting reproducible EI and SA measurements after a morning of training. In contrast, training to effect reproducible CA measurements extended over a month. Figures 1 and 2 demonstrate representative SA measurements for HC, PD, AD, and NPH groups. The red-encoded posterior commissural fibers at the body of the corpus callosum and forceps major were more severely deformed by the gross ventricular distension than the forceps minor and anterior callosal fibers in NPH patients. The SA (Figs. 1 and 2c) is well placed to document these morphological changes, and severely narrowed in NPH patients, in stark contrast to non-NPH subjects. Figure 3 shows the multiple potential pitfalls to positioning a “true” coronal plane and its impact on CA variability. Much effort was expended learning to reformat and position a “true” coronal MPRAGE image at the PC. Specifically, learning anatomical landmarks on the high-resolution 3D MPRAGE sequence (using the internal auditory canal and inner ear structures) to place the coronal image without right-left rotations protracted training. There was greater variability in the CA with erroneous acute/obtuse angulation (Fig. 3a) than with lateral right-left rotations of the coronal plane (Fig. 3b).

**Fig. 4:**
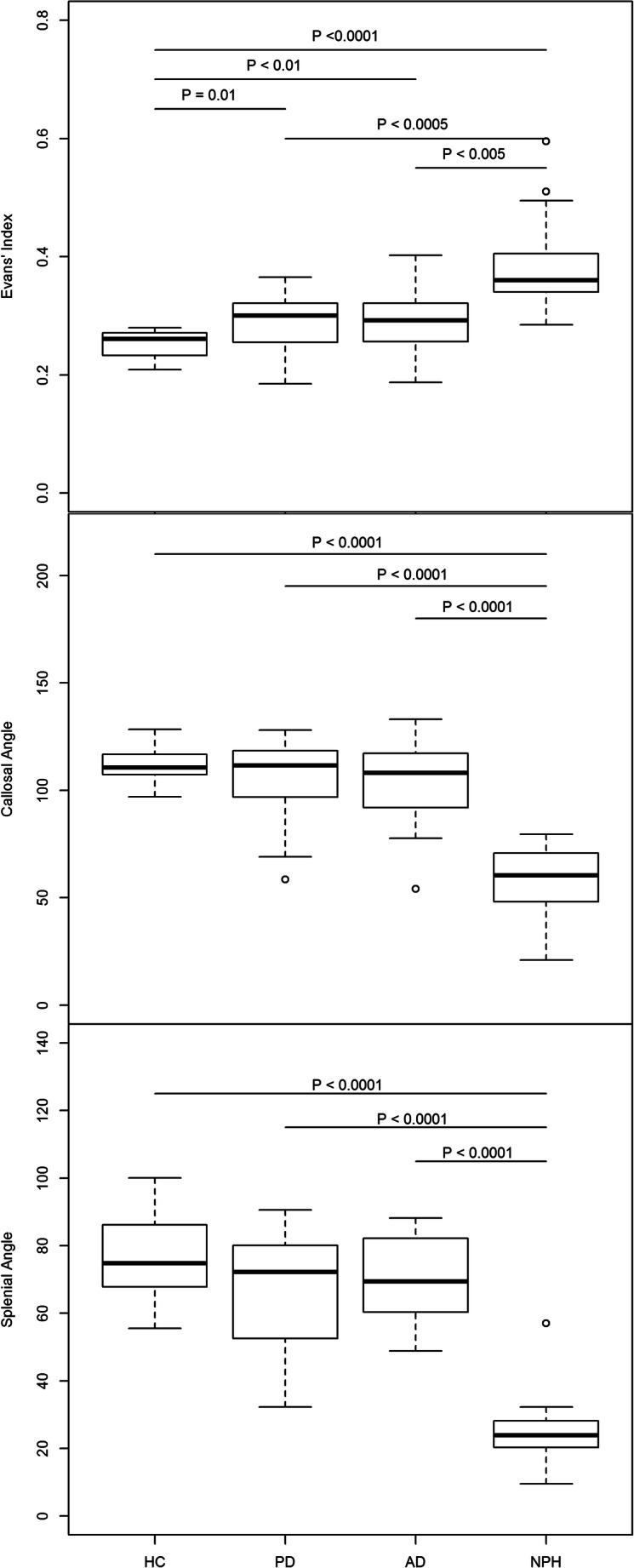
Boxplots of Evans’ index, callosal angle and splenial angle measurements, and pairwise comparisons between healthy controls (HC), Parkinson’s disease (PD), Alzheimer’s disease (AD), and idiopathic normal pressure hydrocephalus (NPH) groups, adjusted by the Benjamini and Hochberg method by controlling the false discovery rate. SA best segregated NPH from non-NPH subjects compared to EI and CA

Pairwise comparisons between groups for EI, CA, and SA are summarized in the box plots in Fig. 4. As expected, only the EI (Fig. 4) differentiated HC from patients, given the established international NPH diagnostic guidelines. There were significant differences (*p* ≤ 0.001) between NPH and non-NPH groups. The SA best segregated NPH from non-NPH groups compared to CA and EI (Fig. 4).

**Fig. 5:**
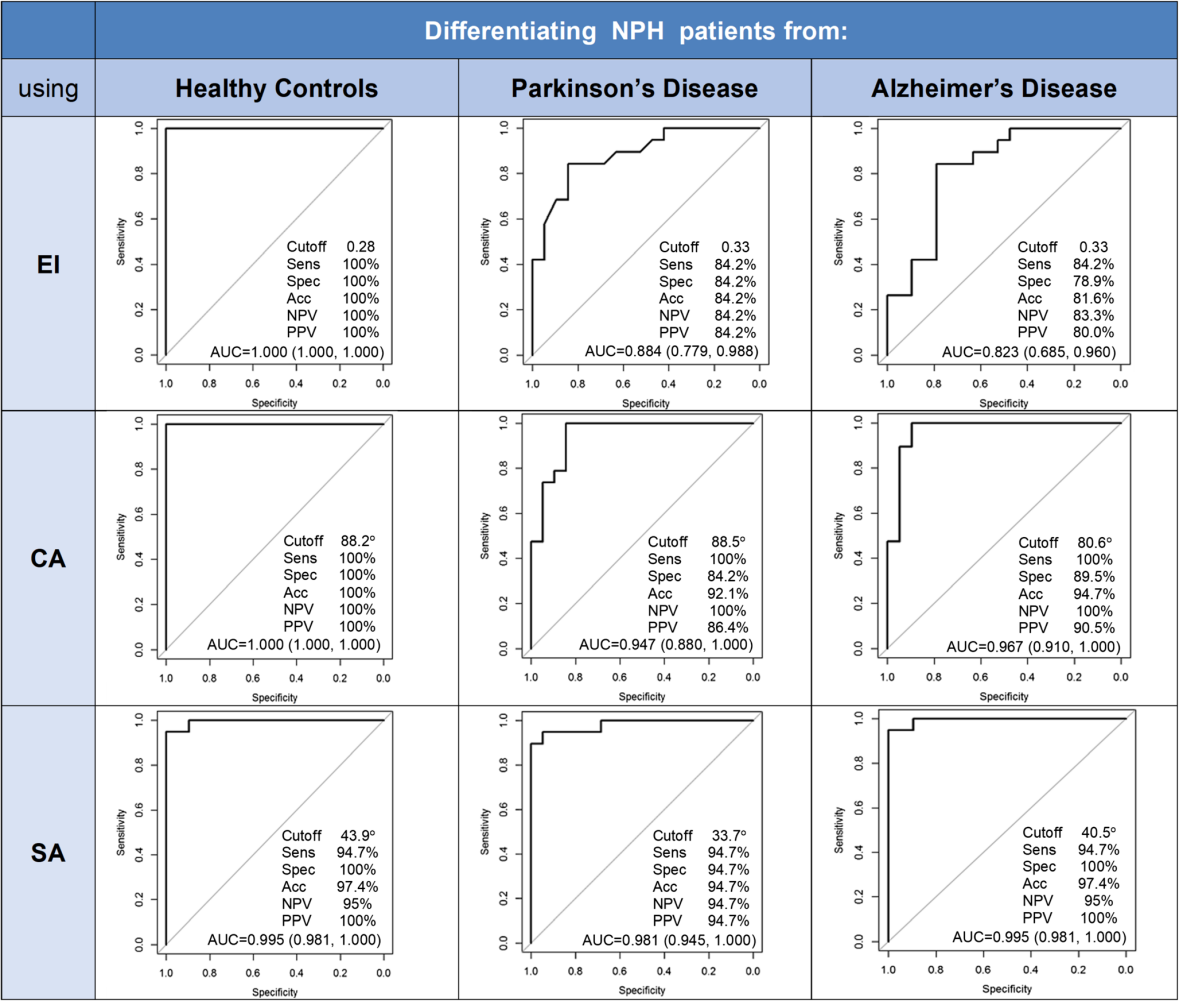
Receiver operating characteristics (ROC) curves for the ability of Evans’ index (EI), callosal angle (CA), and splenial angle (SA) to differentiate idiopathic normal pressure hydrocephalus (NPH) from the healthy control (HC), Parkinson’s disease (PD), and Alzheimer’s disease (AD) groups

The ROC analysis curves for the performance of EI, CA, and SA in differentiating NPH from each non-NPH group individually are detailed in Fig. 5, including cutoff, sensitivity, specificity, accuracy, and positive and negative predictive values. Using cutoff thresholds chosen by the closest to the top-left corner of the ROC plot, SA (AUC > 0.98) performed well in predicting NPH from HC, PD, and AD groups, compared to CA (AUC > 0.91). The cutoffs to identify and distinguish NPH from non-NPH groups of HC, PD, and AD were 43.9°, 33.7°, and 40.5° respectively, with narrower angles favoring NPH. The sensitivities and specificities for these cutoffs were 94.7% and 100%, 94.7% and 94.7%, and 94.7% and 100% respectively. SA was the only measure left in the final model of the multivariable analysis. The addition of more predictors (EI and CA) did not improve the ability to differentiate NPH from the other groups (Table 2).

**Table 2:** Stepwise multivariable logistical regression analysis to predict NPH from non-NPH (HC, PD, AD) groups with brain MRI measures as independent factors

Characteristic	Receiver operating characteristics (95% confidence interval)
EI	0.902 (0.833, 0.971)
CA	0.971 (0.940, 1.000)
SA	0.990 (0.971, 1.000)
SA + EI	0.989 (0.971, 1.000)
SA + CA	0.992 (0.976, 1.000)
EI + CA + SA	0.990 (0.973, 1.000)

*Note: HC* healthy controls, *PD* Parkinson’s disease, *AD* Alzheimer’s disease, NPH idiopathic normal pressure hydrocephalus, *EI* Evans’ index, *CA* callosal angle, *SA* splenial angle

Follow-up brain MRI at 1 year was available in 11 of the 19 NPH patients for further image analysis. Of these, six NPH patients had undergone ventriculoperitoneal shunting shortly after the baseline MRI scan, but five did not due to contraindications to surgery or refusal by patient or caregiver. Table 3 detailed the temporal changes in EI, CA, and SA measurements in these eleven NPH patients. At follow-up MRI, all measures were largely unchanged in non-shunted patients; EI was also stable in shunted patients. Both CA and SA showed similar mean angular widening by 13° at 1-year follow-up. However, the CA variability (± 14.3°) is twice that of SA (± 6.7°), and the angular change more significant for SA than CA measurement given that the baseline SA value was less than half that of CA.

**Table 3:** Temporal changes in brain MRI measures from baseline scan to 1-year follow-up in NPH patients

Change in brain measures between MRI scans 1 year apart	Mean ± SD		*p* value
	With shunt	No shunt	
Evans’ index	−0.03 ± 0.02	0.00 ± 0.01	**0.0294**
Callosal angle (°)	13.2 ± 14.3	−2.1 ± 2.0	**0.0472**
Splenial angle (°)	13.1 ± 6.7	0.2 ± 5.2	**0.0065**

*Note*: Of eleven NPH patients who had follow-up brain MRI at 1 year after baseline MRI, six patients underwent ventriculoperitoneal shunting and five did not. Student’s *t* test was used to compare the changes from baseline in Evans’ index (EI), callosal angle (CA), and splenial angle (SA) between the six NPH patients who underwent shunting and the five who did not, with statistical significance defined at *p* < 0.05 (marked in bold)

## Discussion

In this study, we present the SA, a novel radiological index for the imaging workup of NPH measured on the ubiquitous and conventional axial plane that is standardized in most MRI centers, compared to the less routine coronal plane for CA measurement where the placement of the coronal plane is far more variable between clinical radiological practices and requiring more training to achieve uniformity across sites. The SA is directly determined on the axial DTI color FA maps and easily reproduced with minimal training and without the need for reformatting of the already predefined axial acquisition. The SA is measured using standard radiological tools readily available on the clinical reporting workstation.

DTI sequences are single-shot echo planar acquisitions that are rapidly acquired and very time efficient as an added sequence in the clinical protocol given its unique trajectory, sampling multiple Cartesian lines in k-space in a single readout [10, 11]. The color FA maps indicate the orientation of the major anisotropic white matter fiber tracts and allow easy discrimination of red-encoded commissural tracts traversing the corpus callosum from right to left and green-encoded anterior-posterior cingulate association fibers. With increasing clinical applications of DTI, it may be apt to integrate simple DTI sequences into the routine neuroradiological assessment of NPH. The SA is unique in its sensitivity to ventricular morphological changes and capacity to bypass traditional concerns of imaging markers in NPH, including variability of the CA, complex image post-processing requisites for 3D volumetry or validity of DTI parametric comparisons across sites and scanner makes [2, 4, 7, 9, 13, 15, 19–22]. Our excellent ICC scores for the SA measurement after effortless training attest to its reproducibility.

The CA is variable in untrained hands in routine clinical practice [7, 9, 22]. The CA narrows as the choice of the coronal plane for its measurement moves posteriorly across the corpus callosum [4, 7, 9, 22]. Even when the coronal plane for CA measurement is standardized and fixed at the PC, we found reproducible CA challenging if due training and caution were not undertaken to correct for non-orthogonal or right-left tilting of the said coronal plane (Fig. 3) [9]. These challenges to CA measurements on coronal planes truly orthogonal to the bi-commissural plane contribute to its variable cutoffs reported in the literature. Learning to correct for the erroneous right-left rotation of the coronal plane was time-consuming, albeit the CA variability on these right-left mal-rotated planes were less than those arising from acute-obtuse mal-angulation of the coronal plane. Hence, 3D volumetric data is excellent for fine adjustments to achieve a “true” coronal plane, but this may be difficult to readily translate into busy clinical reporting.

SA outperformed the EI and CA in predicting NPH from each non-NPH group in our ROC analysis (Fig. 5) and predicted NPH from all non-NPH groups with high performance, without improvement after the addition of EI and CA (Table 2). Stark narrowing of the SA in NPH patients reflected severe medial compression and stretching of the posterior callosal fibers and forceps major against the unyielding inferior free edge of the interhemispheric falx secondary to the gross superior expansion and towering of the lateral ventriculomegaly. This is in sharp contrast to the normal fanning of the forceps major and posterior callosal fibers in non-NPH subjects (Figs. 1 and 2). Interestingly, Yamada et al [23] also found linear indices (derived from semi-automated segmentation of ventricular and subarachnoid spaces) defining compression of the brain just above the ventricle useful in differentiating NPH from AD. However, these sophisticated indices require complex image post-processing and analytical tools in 3D quantitative volumetric analysis [23, 24] to enable detailed characterization of ventriculomegaly and distribution of the CSF compartments. Despite their limitations, simple quantitative tools, such as the EI and CA, have been shown to be effective as diagnostic screening tools to differentiate between NPH and non-NPH subjects [4, 7, 22, 25, 26]. The relationship between SA and automated volumetry remains to be determined.

Radiographically, the posterior callosal fibers appear more at risk of deformation by the progressive lateral ventriculomegaly in NPH than the anterior callosal fibers. This may be secondary to blunt pressure from enlarged CSF volumes directed posteriorly against the medial walls of the lateral ventricles as the CSF gush out from the foramen of Monro during diastole. Follow-up MRI in our small NPH subset who underwent shunting showed that the SA was sensitive to temporal morphological changes in post-shunted NPH patients. Perceptible angular changes in both the CA and SA were registered following shunting, suggesting some relief of pressure on the posterior callosal fibers. MRI features in NPH are known to correct partially following shunt intervention [4, 27]. However, similar angular improvement (13°) (Table 3) given the narrower pre-shunting SA (25°) compared to the wider pre-shunting CA (58.3°) (Table 1) suggested that the vertically directed CA measurement might be less effective in capturing the angular change in posterior-medial ventricular wall compression. Of note, the computed angular change was also less variable for the SA than the CA on the follow-up MRI scans, likely reflecting the robustness of the SA measurement. The EI, which documents frontal ventricular pressures and distension, was inadequate in registering the ventricular morphological changes post-shunting, remaining unaffected between shunted and non-shunted NPH patients (Table 3). Meier et al also found the EI to be a poor indicator of clinical response to shunt placement in NPH [6]. The utility of SA as a predictor of shunt responsiveness or indirect index of shunt function warrants further study.

Our study had its limitations. First, the NPH group was compared to AD, PD, and HC datasets that were acquired from non-identical imaging protocols optimized for different studies. Nevertheless, the differences in in-plane resolution for the sequences (DTI/MPRAGE/FLAIR) where SA, CA, and EI were measured were generally < 1 mm^2^. Second, the DTI acquisition times were longer than conventional clinical MR sequences for the purpose of rigorous DTI parametric quantitative analysis in the respective studies. However, the proposed SA is an angular measure that is independent of quantitative DTI parameters and protocol variations [13, 19–21]. A simple DTI sequence with a minimum of six diffusion sensitization non-colinear directions and acquisition time of < 2 min would suffice for generation of useful color-encoded FA maps [10] for SA measurement. Third, we did not compare the efficiency of the SA measurement across other routine axial MRI sequences. In our experience, the body of the corpus callosum in NPH patients is notably compressed and narrowed and we found the 2-2.5-mm slice thickness in the DTI protocol ideal for selection of the axial cut containing the complete body of the corpus callosum for SA placement in NPH patients. Slice selection for SA measurement given partial volume averaging of the thinned-out corpus callosum on 4-5-mm thicker routine conventional T1- and T2-weighted axial imaging would require further assessment.

Future work needs to be done to assess the reproducibility of SA by different raters across sites, its inclusion in radiological scales summarizing imaging features into structured scores for diagnosis or prognosis [28, 29], clinical triaging for further sophisticated 3D volumetric analysis [23, 24] or CSF tap tests for diagnostic confirmation of NPH, and utility as a predictor of shunt responsiveness in a larger cohort of NPH patients undergoing surgical intervention [27]. In addition, while the bi-commissural plane is a common reference standard for axial imaging in many clinical radiological practices and indeed, even for defining the true coronal plane for CA measurements, future work could be done to assess the impact of variations to the central inter-commissural AC-PC line (e.g., the canthomeatal or corpus callosum lines [30] which may be used in other institutions) on the SA values.

## Conclusion

The SA measured on axial DTI color FA maps is a simple radiological index reflecting a horizontal angular perspective of lateral ventricular dilatation in NPH. It shows promise in differentiating NPH from the ex vacuo ventriculomegaly of aging and neurodegenerative disorders and sensitivity to morphological changes before or after shunt surgery in NPH. Further work is needed to evaluate feasibility in translating its efficiency from DTI FA maps to other routine axial CT or MRI sequences.

## Supplementary information

ESM 1 (DOCX 99 kb)

## Abbreviations

AC: Anterior commissure
AC-PC: Anterior-posterior commissure
AD: Alzheimer’s disease
AUC: Area under the curve
CA: Callosal angle
CSF: Cerebrospinal fluid
DTI: Diffusion tensor imaging
EI: Evans’ Index
FA: Fractional anisotropy
FLAIR: Fluid-attenuated inversion-recovery
HC: Healthy controls
MPRAGE: Magnetization-prepared rapid gradient-echo
NPH: Idiopathic normal pressure hydrocephalus
PC: Posterior commissure
PD: Parkinson’s disease
SA: Splenial angle

## Declarations

### Guarantor

The scientific guarantor of this publication is Ling Ling CHAN.

### Statistics and biometry

Dr Huihua Li provided statistical advice for this work and is a co-author in this manuscript.

### Study subjects or cohorts overlap

Some study cohorts have been reported in other prior clinical or unrelated imaging, *viz*, Diffusion Tensor MRI analysis. These were referenced in the manuscript and below:

- Huang X, Ng SY, Chia NS, et al Serum uric acid level and its association with motor subtypes and non-motor symptoms in early Parkinson’s disease: PALS study. *Parkinsonism Relat Disord*. 2018;55:50-4. Epub 2018/05/22. 10.1016/j.parkreldis.2018.05.010. PubMed PMID: 29779681.
- Chan LL, Ng KM, Rumpel H, et al Transcallosal diffusion tensor abnormalities in predominant gait disorder parkinsonism. *Parkinsonism Relat Disord*. 2014;20(1):53-9. Epub 2013/10/16. 10.1016/j.parkreldis.2013.09.017. PubMed PMID: 24126023.
- Prasad K, Wiryasaputra L, Ng A, et al White matter disease independently predicts progression from mild cognitive impairment to Alzheimer’s disease in a clinic cohort. *Dement Geriatr Cogn Disord*. 2011;31(6):431-4. Epub 2011/07/16. 10.1159/000330019. PubMed PMID: 21757908.

### Methodology

• retrospective

• case-control study / cross-sectional study / prognostic study / observational

• performed at one institution
