# Supplementary material for: The splenial angle: a novel radiological index for idiopathic normal pressure hydrocephalus
Source: Eur Radiol. 2021 May 15;31(12):9086–97. doi: 10.1007/s00330-021-07871-4 (PMC8589785; doi:10.1007/s00330-021-07871-4)
Supplement: Supplementary file 1 — (DOCX 99 kb) [file 330_2021_7871_MOESM1_ESM.docx]

**SUPPLEMENTARY MATERIALS**

**Supplementary Figure.** Scatterplot demonstrating correlation between CA and SA in the 4 subject groups, with the R^2^ value for the linear regression of each group indicated at the right lower corner. As can be seen, SA and CA may indicate slightly different aspects of the lateral ventricular distension in NPH, with the SA range narrower (mostly <35^o^) and different from the non-NPH groups (mostly >45^o^) in general.

**
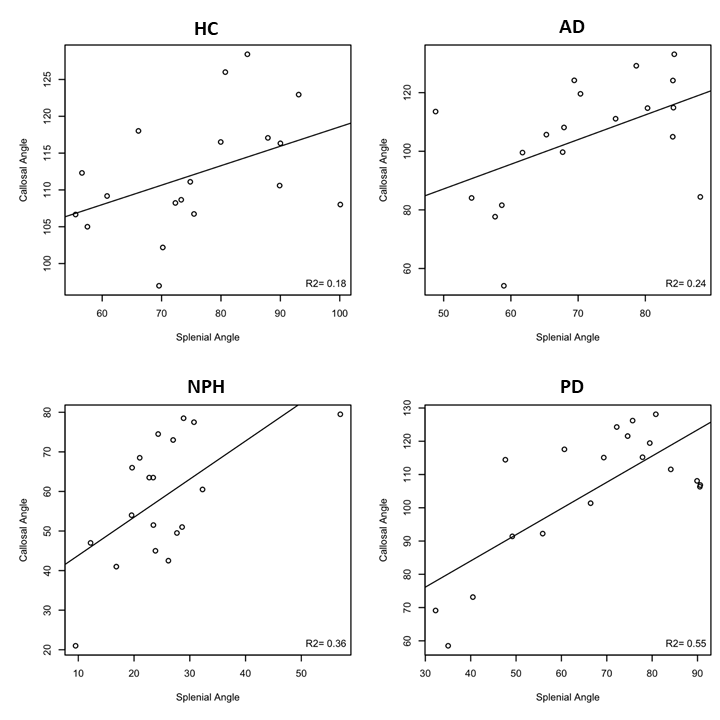
**

| Range of Brain Measures Between Groups | | | | | | | | | |
| --- | --- | --- | --- | --- | --- | --- | --- | --- | --- |
| Group | **HC** | | **PD** | | **AD** | | **NPH** | |  |
| Range | **Min** | **Max** | **Min** | **Max** | **Min** | **Max** | **Min** | **Max** |  |
| EI | 0.21 | 0.28 | 0.19 | 0.37 | 0.19 | 0.40 | 0.29 | 0.60 |  |
| CA (°) | 97.0 | 128.4 | 58.5 | 128.1 | 54.1 | 133.1 | 21.0 | 79.5 |  |
| SA (°) | 55.5 | 100.1 | 32.3 | 90.6 | 48.8 | 88.2 | 9.5 | 57.0 |  |
| *Note:* HC = Healthy Controls, PD = Parkinson’s Disease, AD = Alzheimer’s Disease, NPH = Idiopathic Normal Pressure Hydrocephalus, EI = Evans' Index, CA = callosal angle, SA = splenial angle. | | | | | | | | | |

**Supplementary Table 1.** Minimum and maximum range intervals of quantitative MRI brain measures in NPH and Non-NPH (HC, PD, AD) groups

**Supplementary Table 2.** Intraclass Correlation Coefficients (ICCs) for Inter-Rater Reliability of MRI Brain Measures

| Intraclass Correlation Coefficients (ICC) | |
| --- | --- |
| MRI Brain Measures by Group | **ICC (95% CI)** |
| HC |  |
| Evans’ Index | 0.90 (0.62, 0.97) |
| Callosal Angle | 0.95 (0.89, 0.98) |
| Splenial Angle | 0.97 (0.91, 0.99) |
| PD |  |
| Evans’ Index | 0.92 (0.79, 0.97) |
| Callosal Angle | 0.99 (0.98, 1.00) |
| Splenial Angle | 0.98 (0.94, 0.99) |
| AD |  |
| Evans’ Index | 0.95 (0.30, 0.99) |
| Callosal Angle | 0.98 (0.96, 0.99) |
| Splenial Angle | 0.84 (0.73, 0.96) |
| NPH |  |
| Evans’ Index | 0.98 (0.94, 0.99) |
| Callosal Angle | 0.97 (0.92, 0.99) |
| Splenial Angle | 0.95 (0.83, 0.98) |
| All |  |
| Evans’ Index | 0.97 (0.93, 0.99) |
| Callosal Angle | 0.99 (0.99, 1.00) |
| Splenial Angle | 0.99 (0.98, 0.99) |
| *Note:* HC = Healthy Controls, PD = Parkinson’s Disease, AD = Alzheimer’s Disease, NPH = Idiopathic Normal Pressure Hydrocephalus | |
